# Removal of Heavy Metal Ions from Household Drinking Water Using Acacia Galpinii Seeds and Seed Pods

**DOI:** 10.5696/2156-9614-6.12.7

**Published:** 2016-12-22

**Authors:** Donatus Dube, Candyce Chingoma

**Affiliations:** Department of Applied Chemistry, Faculty of Applied Sciences, National University of Science and Technology, Zimbabwe

**Keywords:** *Acacia galpinii*, adsorption, FTIR, kinetics

## Abstract

**Background.:**

Contamination of drinking water with heavy metals poses a human health threat, particularly in low-income countries where point-of-use water purification systems are beyond the reach of a majority of households.

**Objectives.:**

The study was undertaken to evaluate the efficacy of Acacia galpinii (monkey thorn tree) biomass in removing lead (Pb (ll)), cadmium (Cd (ll)), calcium (Ca (ll)), and magnesium (Mg (ll)) ions from drinking water.

**Methods.:**

A. Galpinii biomass from seed and seed pods was processed by pulverizing, Soxhlet oil extraction and particle size grading. The material was analyzed by X-ray fluorescence (XRF) and Fourier transform infrared (FTIR) spectrophotometry. Influence of the physiochemical parameters (contact time, initial concentration, adsorbent dosage, pH) on the effectiveness of the biomass in removing Pb (ll), Cd (ll), Ca (ll) and Mg (ll) ions was evaluated and the best fit adsorption isotherm model (Langmuir vs. Freundlich) was also determined.

**Results.:**

Particle size, dose, contact time and pH all played significant roles in the effectiveness of metal removal for both seed and seed pod biomass. At biomass particle size <90 microns, 98% removal rates of Pb (II) ions were achieved for powdered seed pods compared with 65% for powdered seeds. The same trend was observed for Cd, Ca and Mg. Contact time for effective removal of metal ions by pod powder and seed powder was 90 minutes and 120 minutes, respectively. Maximum adsorption was achieved at solution pH 6-8 for all metals. Lead adsorption followed a Langmuir isotherm model with maximum adsorption capacities of 10.8932 for pod powder and 3.4412 for seed powder. Adsorption of Ca and Mg followed a Freundlich model, with adsorption capacity of 1.1789 for Ca and 1.4521 for Mg.

**Conclusions.:**

Acacia galpinii seeds and seed pods are inexpensive, readily available and may serve as a cost effective means for treatment of drinking water for domestic users in low and middle income countries.

## Introduction

According to the World Health Organization, an estimated 663 million people worldwide lacked access to safe drinking water in 2014.^1^ Toxic metals such as lead (Pb) and cadmium (Cd) can contaminate drinking water and are associated with a range of adverse health outcomes in humans. According to the United States Environmental Protection Agency and the International Agency for Research on Cancer, many heavy metals are classified as either “known” or “probable” human carcinogens and long term exposure can result in many health complications.^2^,^3^

The potential of lead poisoning, especially in children below the age of six, has been a concern to many environmental agencies across the world, and many governments have banned the use of leaded gasoline, lead paints and soldered cans as a precautionary measure to prevent widespread effects.^3^ According to the US Department of Health and Human Services, lead affects several organs and systems in the body, notably the kidneys and liver, central nervous system, hematopoetic system, endocrine system, and reproductive system.^3^

Cadmium exposure may also cause irreparable damage to body systems, including pulmonary adenocarcinomas.^3^ The International Agency for Research on Cancer and the United States National Toxicology Program have concluded that there is adequate evidence that cadmium is a human carcinogen and some studies have linked it to cancers of the lung, prostate, liver, kidney and gut.^3^,^4^

Although the Millennium Development Goal of halving the proportion of people without access to safe drinking water between 1990 and 2015 has been largely met, globally sectoral challenges remain as under-developed countries, particularly in sub-Saharan Africa, still face enormous challenges.^1^ Rural communities are the furthest from meeting the 2015 Millennium Development Goal's drinking water target. Globally, only 27% of the rural population has water piped directly to their homes and 24% rely on unimproved sources, i.e. directly from dams, unprotected wells, rivers, etc. Of the 663 million people without access to an improved water source globally, 319 million (48%) live in rural areas of Sub-Saharan Africa which has made the least progress in improving sources of drinking water since 1990, improving by only 20% as of 2014. In contrast, the Eastern Asian region saw a dramatic drop from 45% to below 9% reliance on unimproved water sources in the same time period.^1^ Sachs et al. argue that poor communities often lack access to potable water and the provision of improved sources of water is linked to their ‘transition out of poverty’.^5^

Drought is a common problem in Zimbabwe, particularly in the southern provinces of Matabeleland and Masvingo. Although many boreholes have been sunk, they often dry up because of drought and the lack of funds and spare parts to maintain the pumps; moreover, water from some of the boreholes is of unacceptable quality as it contains high levels of toxic heavy metals.^1^,^6^

The quest for alternative water treatment methods that use low cost materials such as clay, zeolites, coal, fly ash, peat, siderite, agricultural wastes, charcoal and naturally occurring adsorbents has generated much interest. Some toxic heavy metals can be removed from water using plant and non-plant adsorbents.^7–9^ For example, work has been done with various plant-based biomasses of which Eicchorniacrassipes, reeds, sunflower stems, sawdust, bael fruit and wheat straw have proved to be effective biosorbents.^10^,^11^ Other studies have used dried beans, peach seeds, and Moringa oleifera seeds to treat water to acceptable water quality levels.^12^,^13^ Most of this work has, however, been limited to laboratory practice and more needs to be done to get the technologies to where they are needed most, in rural communities.

**Abbreviations**
*Ca*: Calcium
*Cd*: Cadmium
*FTIR*: Fourier transform infrared
*Mg*: Magnesium
*Pb*: Lead
*XRF*: X-ray fluorescence

We conducted a series of batch adsorption experiments to evaluate the efficacy of Acacia galpinii (monkey thorn tree) biomass in removing Pb (ll), Cd (ll), calcium (Ca (ll)) and magnesium (Mg (ll)) ions from water. These ions are particularly problematic in underground water in many parts of the country and are strictly monitored by the Zimbabwe Environmental Management Agency. A. galpinii is abundant in Zimbabwe, particularly the southwestern parts of the country, and seasonally produces seeds, and hence also seed pods in abundance. In particular, we investigated the influence of physiochemical parameters such as contact time, initial concentration, adsorbent dosage and pH on the removal of heavy metals. Our overall objective was to provide proof-of-concept data on this rudimentary potential point-of-use water treatment method for rural areas of Zimbabwe.

## Methods

### Sample Collection

Dry A. galpinii pods containing seeds were collected from trees along Cecil Avenue, Ascot Suburb in Bulawayo, Zimbabwe from January–February 2015. The pods were separated manually and classified visually into large, medium and small. Large pods were then selected for the study assuming that they were more likely to contain the most mature seeds.

The seeds were manually separated from the pods, and healthy looking seeds selected for the study. Selected seeds and pods were washed with distilled water and sun dried for two days before oven drying at 50°C for 8 hours. Dried seeds and pods were then ground separately into fine powder using a standard laboratory blender with stainless steel blades. The seed and pod powders were sieve graded into <90, 90–125, 125–179, 180–249, 250–424 and >424 μm fractions using a sieve shaker.

Soxhlet extraction was used to extract oils from the seed and pod powders. Briefly, 10 g of powder was placed into an extraction thimble, while 170 ml of hexane solvent was poured into a round-bottomed flask. The hexane was heated for 45 minutes to extract the oil. The residue was rinsed with diethyl ether to remove traces of oil and the defatted powder oven dried at 50°C for 24 hours to completely drive out moisture.^14^

The elemental composition of the dried biomass was determined at Hwange Colliery Company, Zimbabwe, using X-ray fluorescence (XRF) spectrometry following Hossain et al. (2012), and the major functional groups were determined at Midlands State University, Gweru, Zimbabwe, using Fourier transform infrared (FTIR) spectrometer (FTIR-8400S, Shimadzu, Singapore) at wavenumber 400–4000 cm^−1^.^15^

Next, 10 mg/L individual solutions of Mg, Ca, Cd and Pb were prepared from 1000 mg/L stock solutions prepared from the metal salts and used in the subsequent experiments.

### Analytical Methods

Water quality parameters were measured following standard procedures suggested in the Standard Methods for the Examination of Water and Wastewater.^16^ For the present study, Pb, Mg, Cd and Ca were measured at the National University of Science and Technology, Bulawayo laboratory using a Spectra 20 (Varian Instruments, Palo Alto, California, USA) atomic absorption spectrometer.^17^ Prior to analysis, the atomic absorption spectrometer was calibrated using commercial standard samples from Glassworld, Robertville, South Africa. Next, pH and conductivity were measured using a Multi-parameter Testr 35 Series (Eutech Instruments, Ayer Rajah Crescent, Singapore).

All experiments were conducted in triplicate and mean values were reported.

### Adsorption Experiments

#### Dosage Experiments

Various masses of seed and pod powders (0.05 g, 0.1 g, 0.15 g, 0.2 g and 0.25 g) were added to 50 ml of the 10 mg/L metal solutions (Pb, Mg, Ca and Cd). The resulting mixture was agitated at 240 rpm for 90 minutes and filtered using a Buchner funnel. The filtrate was analyzed for the target metals as described above.

The amount of metal adsorbed by the adsorbent at equilibrium (q_e_) was calculated using Equation 1.

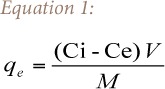
where Ci and Ce are the initial and equilibrium concentrations in mg/L of the metal ions respectively, V is the volume in liters of the solution and M is the mass in grams of the adsorbent.

#### Contact Time Experiments

An amount of 50 ml of each 10 mg/L metal solution was added to 0.1 g of seed and pod powder. After agitation at 240 rpm, aliquots were withdrawn at 20 minutes, 40 minutes, 60 minutes, 90 minutes and 120 minutes, and filtered. The residual metal concentration was quantified as described and the amount of metal adsorbed calculated using Equation 1.

#### Particle Size Experiments

A total of 0.1 g of seed and pod powder was taken from <90, 90–125, 125–179, 180–249, 250–424 and >424 μm size fractions and added to 50 ml of 10 mg/L metal solution. The mixture was agitated, filtered and analyzed as described previously and the amount of metal adsorbed calculated using Equation 1.

#### pH Experiments

Solutions of differing pH were prepared by adjusting the pH of a 50 ml, 10 mg/L metal solution using 0.1 normal sodium hydroxide or 0.1 normal nitric acid. Next, seed or pod powder was added to the resulting solution and agitated, filtered and analyzed for metal concentrations as previously described. The amount of metal adsorbed was calculated using Equation 1.

#### Batch Adsorption Experiments

Portions of 0.1 g Acacia galpinii seed and pod were weighed into 250 ml beakers to which 50 ml of sample solution with varying initial metal ion concentrations between 10–50 mg/L were added at ambient temperature. The samples were then agitated for 90 minutes. The suspensions were filtered using Whatman No. 1 filter paper. The filtrates were collected in separate clean bottles and metal content was determined using an atomic absorption spectrometer. The percent (%) metal ion absorbed was calculated using Equation 2.

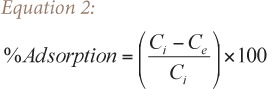
where C_i_ and C_e_ are the initial and final concentrations of the metal ions in solution, respectively.

#### Adsorption Isotherms

The experimental data was fitted into two adsorption models, Langmuir and Freundlich. The Langmuir isotherm was developed based on the assumption that each site can accommodate only one molecule of the adsorbate with no molecule migration and the energy of adsorption is constant all over the surface.^18^ The linear form of the model is shown in Equation 3, where q_e_ is the equilibrium metal ion concentration on the adsorbent (mg/g), q_max_ is the maximum adsorption capacity on monolayer adsorbent saturation (mg/g), C_e_ is the equilibrium metal ion concentration in solution (mg/L), and b is the Langmuir affinity constant (L/mg). A plot of 1/q_e_ versus 1/C_e_ for each metal ion was made.


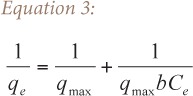


The Freundlich isotherm was developed so as to model the multilayer adsorption on heterogeneous surfaces (Freundlich, 1906). The linear form of the model is shown in Equation 4, where q_e_ is the amount of metal ion adsorbed at equilibrium (mg/g), C_e_ is the concentration of the adsorbate at equilibrium (ppm), k_f_ is the Freundlich constant related to adsorption capacity and n is a dimensionless heterogeneity coefficient. A plot of lnq_e_ against InC_e_ for each metal was also made.


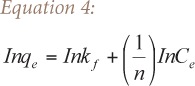


## Results

### Biomass Characterization

The FTIR spectra of seed and pod powder is shown in the Supplemental Material. The spectra shows the following peaks: 1) 3423–3463 cm^−1^ (typical of those found in proteins, fatty acids, carbohydrates and lignin units); 2) 1639–1639 cm^−1^ (carbonyl group); 3) 1037–1049 cm^−1^ (methoxy group); and 4) 2923 cm^−1^–2926 cm^−1^ (methanetriyl group bond stretching).

Table 1 summarizes the XRF-derived elemental composition of pod and seed powder, showing the predominance of calcium oxide, silicon dioxide, phosphorus pentoxide, potassium oxide and calcium oxide in both biomasses.

**Table 1:** XRF Results (in mg/L) for Pod and Seed Powders

***Compound***	**Pod Powder**	**Seed Powder**
**Silicon dioxide**	0.536	3.223
**Titanium dioxide**	−0.002	−0.001
**Aluminum oxide**	0.278	1.286
**Iron(III) oxide**	0.135	0.179
**Calcium oxide**	3.985	2.674
**Magnesium oxide**	0.476	1.094
**Potassium oxide**	0.829	4.221
**Sodium oxide**	0.267	0.681
**Chromium(III) oxide**	0.022	0.023
**Phosphorus pentoxide**	0.286	2.446
**Sulfur trioxide**	0.680	4.330
**Phosphorus**	0.055	0.508
**Sulfur**	0.120	0.940

### Experimental Results

**Figure 1:**
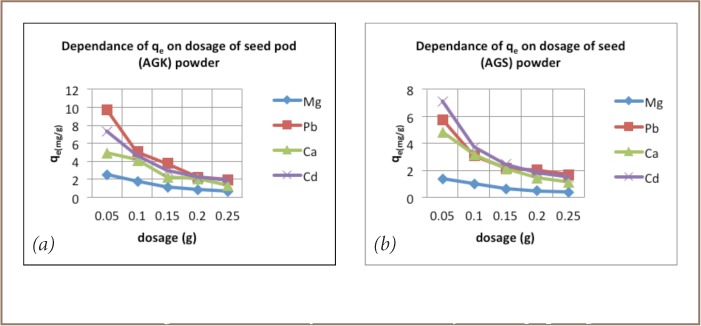
Amount of metal adsorbed by Acacia galpinii pod (a) and seed (b) powders at equilibrium (q_e_) as a function of powder dosage.

The dosage experiments showed a decrease in equilibrium metal ion concentration adsorbed on the adsorbent (q_e_) as we increased the amount of biomass in the solution (*Figure 1*).

**Figure 2:**
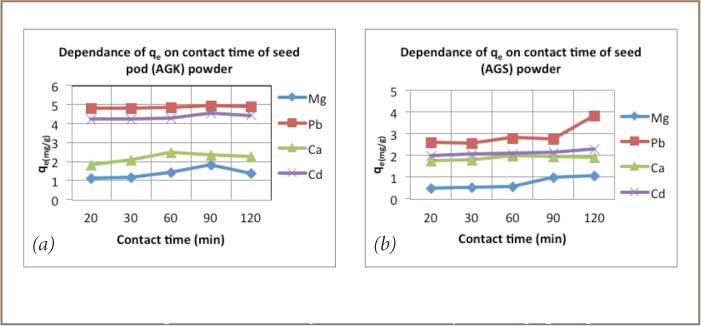
Amount of metal adsorbed by Acacia galpinii pod (a) and seed (b) powders at equilibrium (q_e_) as a function of powder dosage.

The contact time experiments showed that optimum contact time for effective removal of all metal with pod biomass was 90 minutes, while that for seed powder was 120 minutes (*Figure 2*).

**Figure 3:**
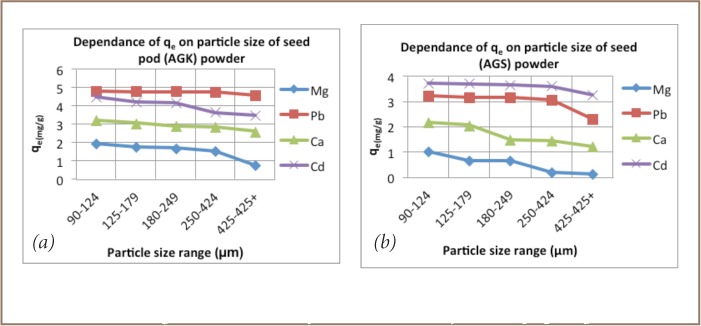
Amount of adsorbed metal by Acacia galpinii pod (a) and seed (b) powders at equilibrium (q_e_) as a function of particle size.

The particle size experiments showed that removal of metals by both biomasses is dependent on particle size of the adsorbent, with finer material generally exhibiting higher effectiveness (*Figure 3*).

**Figure 4:**
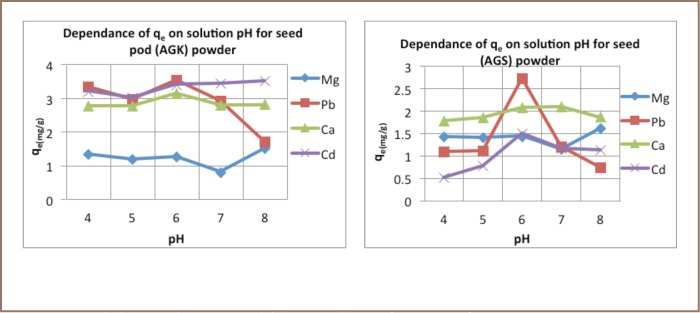
Amount of adsorbed metal by Acacia galpinii pod (a) and seed (b) powders at equilibrium (q_e_) as a function of pH.

The pH experiments showed that maximum adsorption was achieved at pH 6–8 for both biomasses and all metals investigated (*Figure 4*).

**Figure 5:**
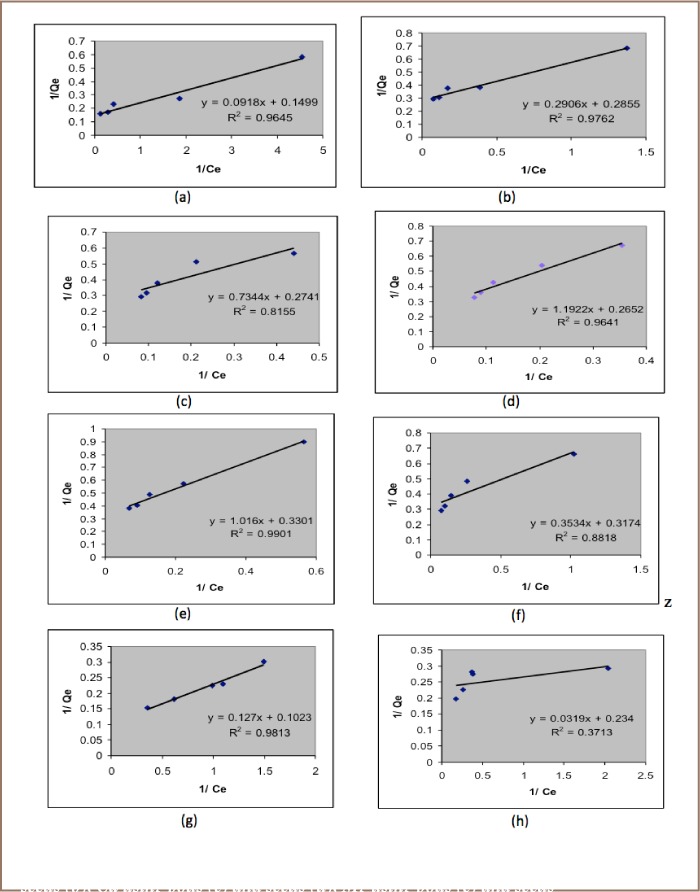
Langmuir adsorption isotherms for Pb using pods (a) and seeds (b); Ca using pods (c) and seeds (d); Mg using pods (e) and seeds (f); Cd using pods (g) and seeds (h).

**Figure 6:**
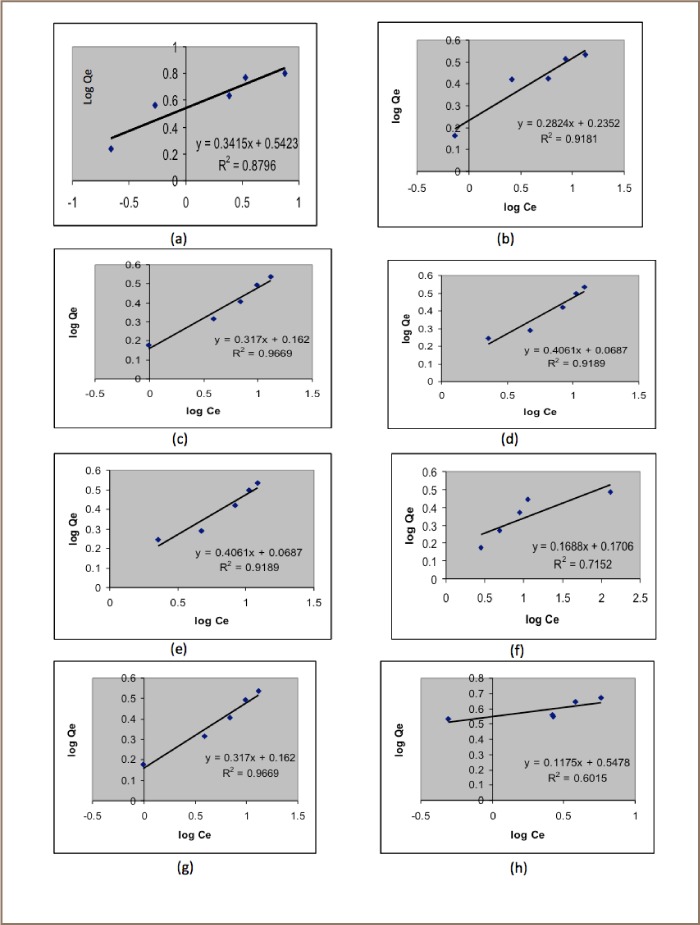
Freundlich adsorption isotherms for Pb using pods (a) and seeds (b); Ca using pods (c) and seeds (d); Mg using pods (e) and seeds (f); Cd using pods (g) and seeds (h).

Figure 5 presents the Langmuir isotherms for the target metals and Figure 6 presents the Freundlich isotherms.

Lead adsorption using pod and seed best fit the Langmuir isotherm. The maximum adsorption capacities were 10.8932 for pod and 3.4412 for seed. The R_L_ value, a dimensionless constant referred to as separation factor or equilibrium parameter, was found to be 0.08 for pod and 0.05 for seed. The R_L_ value indicates the adsorption nature to be either unfavorable if R_L_>1, linear if R_L_ =1, favorable if 0< R_L_<1 and irreversible if R_L_=0. From the data calculated in Table 3, the R_L_ is greater than 0 but less than 1, indicating that the Langmuir isotherm is favorable.

Adsorption of Ca using pod best fit the Freundlich isotherm model with an R^2^ value of 0.9189 and an adsorption capacity of 1.1789. R^2^ is a statistical measure of how close the data are to the fitted regression line. When R^2^=1 (or very close to 1) then the data fits well with the isotherm model. However, the adsorption for Ca using seed powder was best suited to the Langmuir isotherm with a R^2^ value of 0.9641 and an adsorption capacity of 0.8388. The R_L_ value was found to be 0.02.

The adsorption of Mg using pod powder best fit the Freundlich isotherm with an R^2^ value of 0.9669 and an adsorption capacity of 1.4521, and Mg adsorption in seed powder best fitted the Langmuir isotherm with an R^2^ value of 0.9901 and an adsorption capacity of 0.9843.

Cadmium adsorption in both pod and seed powders fit neither the Langmuir nor Freundlich model, as the R^2^ in both cases was very low.

## Discussion

The results of this study confirm several trends. First, a number of adsorption peaks were revealed, confirming the complex nature of the studied adsorbent (*Supplemental Material*). The seed material was found to contain free and intermolecular bonded hydroxyl groups (3423–3463 cm^−1^ peak) typical of those found in proteins, fatty acids, carbohydrates and lignin units. The carbonyl group (−C=O) (1639–1639 cm^−1^ peak) and methoxy group (−OCH_3_) (1037–1049 cm^−1^ peak) also confirmed the presence of the lignin units, while stretching of the methanetriyl group (C-H) bond of the methylene group (CH_2_) was noticed in the wavelength range of 2923 cm^−1^ - 2926 cm^−1^.^15^,^19^ The seed pod spectrum appeared similar. The chemical composition (*Table 1*) shows that the predominant oxides in the material were calcium oxide (for pods) and silicon dioxide, phosphorus oxide, potassium oxide and calcium oxide (for seeds), which is typical for many kinds of biomass.^20^

Second, seed pods were more effective at removing metals than seeds (*Figures 1–3*). For example, pods achieved 97% removal of Pb at particle size 90–124 μm, while seed material only managed to remove 63% under the same conditions.

Third, particle size, dose, contact time and pH all played significant roles in the effectiveness of metal removal. The finer the material, the greater the surface area and more effective the removal of metal ions.^15^ Increasing the dose beyond 2 g/L did not result in any marked increase in metal ion reduction. Student's t-test confirmed 2 g/L as the optimum dosage, thus any increase would likely be a mere waste of material.^8^,^21^ Contact time for effective removal of metal ions by pods and seeds was 90 minutes and 120 minutes, respectively. Solution pH also had a marked effect on adsorption capacity. Except for Ca and Mg, with maximum adsorption at pH 7 and 8 respectively, the rest of the ions reached maximum adsorption at pH 6 (*Figure 4*), in line with previous studies on Moringa oleifera and Moringa stenopetala, demonstrating that medium pH values tend to result in lower electrostatic repulsion between the metal cations and the adsorption surface.^21^

The batch adsorption studies we conducted yielded positive results for Pb, Ca and Mg, but were inconclusive for Cd. Lead adsorption followed a Langmuir isotherm (*Figure 5*), with maximum adsorption capacities of 10.8932 for pod powder and 3.4412 for seed powder. R_L_ values were 0.08 for pod and 0.05 for seed, indicating that adsorption using both adsorbents is favorable (R_L_<1). Pb adsorption can thus be best described as predominantly monolayer sorption onto surfaces with unlimited number of identical sites.^22^ Adsorption of Ca and Mg, on the other hand, followed a Freundlich model, with an R^2^ value of 0.9189 and an adsorption capacity of 1.1789 for Ca and R^2^ value of 0.9669 and an adsorption capacity of 1.4521 for Mg (*Figure 6*). This is best described as a non-ideal and reversible multilayer sorption with a non-uniform energetic distribution of active sites, and/or interactions between sorbed species.^23^ Cd adsorption did not fit either model and other models were not tested.

Due to equipment and method limitations, we did not conduct desorption or column studies, which may have provided useful data on effects of backwashing and continuous adsorption on the effectiveness of the adsorbent material. Regardless, our results are consistent with findings using different types of biomass.^8–10^,^13^,^14^

## Conclusions

Overall, Acacia galpinii (monkey thorn tree) pod and seed powders are promising alternative absorption agents for removal of metals from drinking water. Acacia galpinii pod and seed powders are inexpensive, readily available and may serve as a cost effective means for treatment of drinking water for domestic users in low- and middle-income countries. The advantage of the residue from biomass over residues from other water treatment methods like reverse osmosis, coagulation and sedimentation and ultra-filtration is the ease of its disposal. Future research is needed to investigate the effectiveness of pod and seed in a continuous flow system, including the design, development and testing of a prototype. With further development, there is a potential for providing a cheap, effective heavy metal removal method for rural and suburban water users in poor communities. There is also a need to test the method on other heavy metals like arsenic and mercury which could be problematic in other parts of the world such as parts of South East Asia, South America and Africa.

The method, however, poses some notable obstacles, particularly in the preparation of the seeds and pods for use as an adsorbent material. Extraction of oils can pose challenges as solvents like hexane and diethyl ether can be difficult to obtain, are expensive and hazardous, and require additional investment in material and training of users. Furthermore, holding of ground adsorbent for long hours at 50°C may be challenging, requiring extra care from users. Preparation of proper dosage cups will be required to minimize errors by users in local communities.
