# Supplementary material for: Removal of Heavy Metal Ions from Household Drinking Water Using Acacia Galpinii Seeds and Seed Pods
Source: J Health Pollut. 2016 Dec 22;6(12):7–14. doi: 10.5696/2156-9614-6.12.7 (PMC6221502; doi:10.5696/2156-9614-6.12.7)
Supplement: Supplementary file 1 [file Dube_SuppMaterial.docx]

###

Supplemental Material

Fourier Transform Infrared Spectrograph of *Acacia galpinii* seed (a) and pod (b) powders at wave
number 400–4 000 cm^-1^.

###
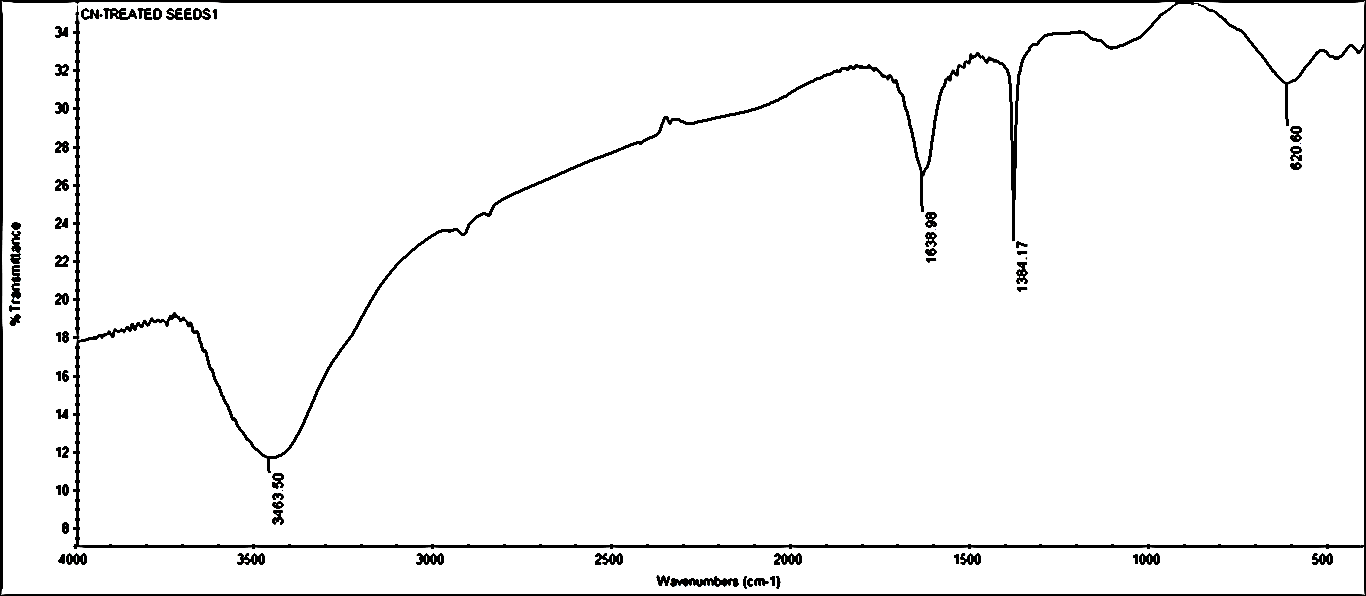

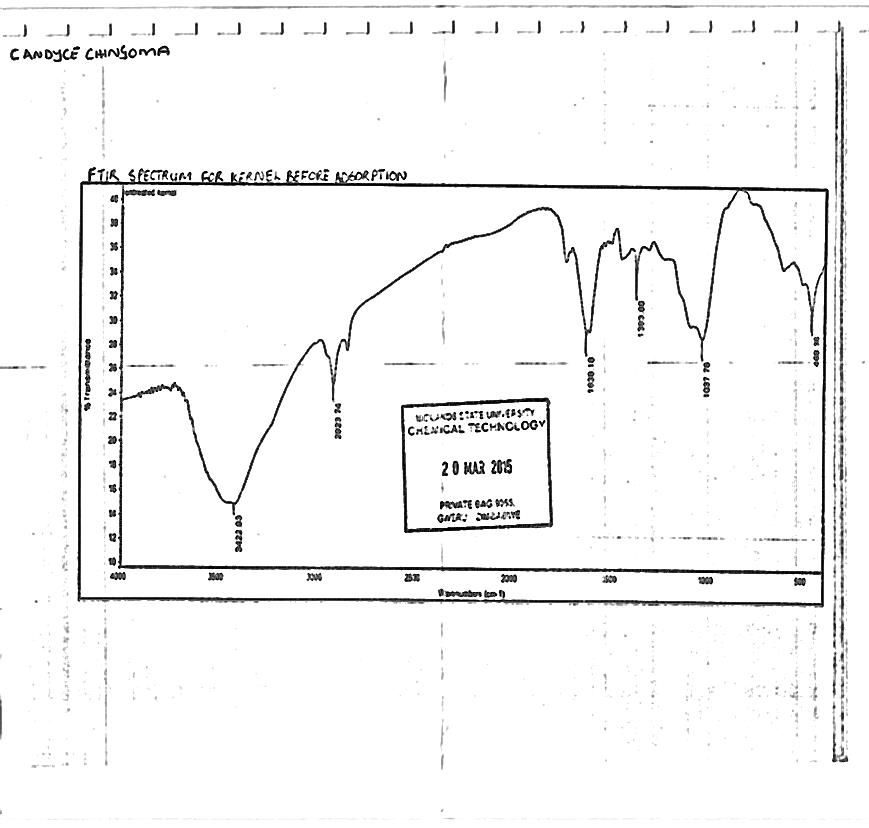


a)

b)
